# Aerobic physical activity and resistance training: an application of the theory of planned behavior among adults with type 2 diabetes in a random, national sample of Canadians

**DOI:** 10.1186/1479-5868-5-61

**Published:** 2008-12-02

**Authors:** Ronald C Plotnikoff, Kerry S Courneya, Linda Trinh, Nandini Karunamuni, Ronald J Sigal

**Affiliations:** 1 Centre for Health Promotion Studies, School of Public Health, University of Alberta, Edmonton, Alberta, Canada; 2 Faculty of Physical Education and Recreation, University of Alberta, Edmonton, Alberta, Canada; 3 School of Public Health, University of Alberta, Edmonton, Alberta, Canada; 4 Department of Medicine, Cardiac Sciences and Community Health Sciences, Faculty of Medicine and Kinesiology, University of Calgary, Calgary, Alberta, Canada

## Abstract

**Background:**

Aerobic physical activity (PA) and resistance training are paramount in the treatment and management of type 2 diabetes (T2D), but few studies have examined the determinants of both types of exercise in the same sample.

**Objective:**

The primary purpose was to investigate the utility of the Theory of Planned Behavior (TPB) in explaining aerobic PA and resistance training in a population sample of T2D adults.

**Methods:**

A total of 244 individuals were recruited through a random national sample which was created by generating a random list of household phone numbers. The list was proportionate to the actual number of household telephone numbers for each Canadian province (with the exception of Quebec). These individuals completed self-report TPB constructs of attitude, subjective norm, perceived behavioral control and intention, and a 3-month follow-up that assessed aerobic PA and resistance training.

**Results:**

TPB explained 10% and 8% of the variance respectively for aerobic PA and resistance training; and accounted for 39% and 45% of the variance respectively for aerobic PA and resistance training intentions.

**Conclusion:**

These results may guide the development of appropriate PA interventions for aerobic PA and resistance training based on the TPB.

## Background

The most recent national surveillance of diabetes prevalence indicates 5.1% of Canadian adults were living with diagnosed diabetes in 1999 [1], with 90% of these diagnoses being type 2 diabetes (T2D) [1]. The prevalence is continuing to rise [2]. In the United States, 9.6% of adults age 20 years and older have diabetes [3]. Given the increasing prevalence of diabetes in the population, there have been calls for the development of effective prevention and treatment strategies [4-6].

Physical activity (PA) plays a central role for both the treatment and prevention of diabetes [7]. Aerobic PA greatly decreases the risk of developing T2D [8] and reduces the risk of progressing from impaired glucose tolerance to T2D [9,10]. For those living with T2D, aerobic PA enhances insulin sensitivity [11-14], improves blood glucose control [15,16], and produces favourable changes in body composition [17]. Therefore, aerobic PA is a well-established recommendation for those with, and at risk for T2D [7,18]. Despite the widespread promotion of aerobic activity however, less than 30% of the diabetic population participate in aerobic PA enough to meet the recommended guidelines of 150 minutes/week of moderate PA [1], which is less than the general population [19].

Recent literature has depicted resistance training as a promising avenue for individuals with diabetes [20]. Progressive resistance training, in which the resistance against the muscles is gradually increased over time, leads to gains in muscle mass [21]. Similar to aerobic PA, resistance training also substantially improves insulin sensitivity [22], and glycemic control [23-27]. Furthermore, performance of both aerobic and resistance exercise was superior to either type of exercise alone for improvement in blood glucose control [28]. Despite the resistance training recommendations implemented by the American College of Sports Medicine [21], American Diabetes Association [7], and the Canadian Diabetes Association [1], the majority of adults with T2D do not perform this behavior [4]. In a population-based assessment of PA levels in 1,193 adults with T2D, Plotnikoff [4] reported only 12% were weight training or performing activities that would increase muscular strength.

The encouragement of inactive individuals with T2D to be active is the first step in PA treatment, and between the two modes of PA, resistance training may be a more attractive option than aerobic activities for some of this population [4]. This is because a significant number of adults with T2D have difficulty with aerobic exercise due to excess weight and/or foot ailments [29]; such individuals might have less difficulty with resistance training [20]. Further, for some individuals, resistance training may be less daunting psychologically than a 30-minute walk, as this population likely associates aerobic PA with shortness of breath, fatigue, and possibly pain [4]. In addition, relative increases in strength through resistance training are typically achieved more quickly, and are of greater magnitude, than increases in aerobic fitness through aerobic PA. This could cause resistance training to be more gratifying, and consequently more motivating than aerobic activity. Furthermore, success at resistance training might lead individuals to be more comfortable with the idea of beginning or increasing aerobic activities.

Implementing theory-based interventions are more effective at changing health-related behavior than atheoretical approaches [30]. However, prior to the translation of theories into interventions, theories and their assumptions should be empirically tested to ensure adequacy. Social cognitive theories such as the *Theory of Planned Behavior (TPB) *[31] are helpful for explaining PA behavior [32-34].

The TPB proposes that a person's *intention *to perform a behavior is the immediate proximal predictor of that behavior since it reflects the level of motivation a person is willing to exert to perform the behavior [31]. Intention is theorized to mediate the influence of three main constructs on behavior: attitude, subjective norm, and perceived behavioral control (PBC). Attitude reflects a positive or negative evaluation of performing the behavior, and has both instrumental (e.g., harmful/beneficial) and affective (e.g., boring/enjoyable) components. Subjective norm is defined as the perceived social pressure to perform the behavior, and includes both *injunctive *(e.g., what significant others think the person ought to do) and *descriptive *(e.g., what significant others themselves do) components. PBC is an evaluation of how easy or difficult it will be to perform a behavior. Application of the TPB identifies underlying beliefs that determine one's attitude, subjective norm and PBC [31], and can provide an understanding of the factors that help initiate behavior (such as PA) for the promotion of related programs to a target group in the population [35,36].

Structured reviews [37] and meta-analyses [33,38] have demonstrated that the TPB is useful for the prediction of health behaviors. In a review of health behaviors conducted by Armitage and Conner [37], attitude, subjective norm and PBC accounted for 40% of the variance in intention, while intention and PBC accounted for 27% of the variance in behavior. Similar results have been reported in a meta-analysis for the PA domain [32,33].

Only one previous study has examined TPB to explain aerobic PA in the diabetic population [34]. In this study by Plotnikoff and colleagues, the TPB constructs were tested in predicting aerobic PA over a six-month period in a large population sample of 697 type 1 and 1614 type 2 adults. The researchers found for both diabetes types across both cross-sectional and longitudinal analyses, attitudes, subjective norm and PBC were all significantly associated with intention, and intention was significantly associated with behavior. PBC was directly related with 6-month PA, but this association was not significant in the cross-sectional analysis. We found only three published studies that have examined the psychosocial predictors of resistance training, all of which assessed adults without diabetes. Of these studies, two tested the TPB [39,40], while the other study [41] examined psychological factors. Dean et al. [40] tested the efficacy of the TPB to explain strength training in older adults (N = 200) and found that subjective norm and PBC explained 42% of the variance in intention and intention explained 40% of the variance in behavior. Bryan and Rocheleau [39] examined the predictive validity of the TPB for aerobic-exercise behavior versus resistance training among a sample of 210 college students and found TPB variables, extroversion, and perceived health collectively accounted for 19% and 40% of the variance in aerobic and resistance exercise respectively. Jette et al. [41] explored the factors associated with exercise participation and adherence to a home-based resistance training in a sample (N = 102) of older people representing a range of functional limitations. The findings revealed that psychosocial factors such as positive attitudes and sense of control toward exercise were important predictors of adherence [41].

In summary, resistance training is beneficial in T2D and there are reasons to believe that TPB might be salient for the prediction of this behavior. Also, no previous study has simultaneously examined the cognitive predictors of aerobic PA and resistance training within a randomly selected population sample. It is imperative that psychosocial determinants for both aerobic PA and resistance training are understood in order to guide the development and tailoring of effective and efficacious programs for the diabetes population. The purpose of this study was therefore to investigate the utility of the TPB in explaining the behaviors of aerobic PA and resistance training in a population sample of T2D adults – our main study objective. A secondary objective was to compare the mean scores of the TPB constructs between aerobic PA and resistance training.

## Methods

### Sampling Frame, Recruitment and Sample

A random digit dialing protocol was employed to recruit individuals with T2D. A random national sample was created by generating a random list of household phone numbers (a national diabetes registry does not exist in Canada). The list was proportionate to the actual number of household telephone numbers for each Canadian province (with the exception of Quebec which had a lesser proportion of phone numbers as predominately Francophone speaking communities were excluded from the sampling frame).

A pretest (n = 7) was conducted to refine the questionnaire and to check the interview length, question wording and interview instructions. The baseline (time 1) questionnaires were mailed in March 2006. The time 2 follow-up questionnaires were mailed in June 2006 employing a rolling mailout pattern to ensure three months had passed between time 1 and time 2. This research was reviewed and approved by local Research Ethics Boards, and all subjects gave informed consent.

### Measures

Socio-demographic factors were measured using questions based on Statistics Canada 2001 census [42] and included: age, gender, marital status, ethnic affiliation, education, income levels, internet access, current health conditions (i.e., angina, heart attack, stroke, cancer, high cholesterol, high blood pressure) smoking status, and the age the participants were diagnosed with type 2 diabetes. These measures have been employed by our research team in previous studies [34,43,44].

PA behavior was assessed using a modified version [19] of the validated Godin Leisure-Time Exercise Questionnaire (GLTEQ) [45,46]. For aerobic activity, participants were asked to report the average number of times per week and average duration in the past month they engaged in strenuous, moderate, and mild intensity physical activity for a minimum of 10 minutes per session. Occupational and household activities were not included. Participation responses for the strenuous and moderate activity categories in each activity category were then added to obtain a summary score of the number of minutes of physical activity per week (mild intensity activity responses were not included in the calculation). A resistance training item was added to the GLTEQ where participants were asked to report the average number of times per week and average duration in the past month they engaged in resistance training. The summary score for resistance training behavior was computed by multiplying the average frequency and average duration in the past month.

### Social-Cognitive Variables

Aerobic PA-related measures that were initially developed for a previous study [34] examining aerobic activity were employed for this study. With regards to the resistance training TPB measures, we modified the aerobic TPB items through a process which included cognitive interviews. After the cognitive interviews were conducted (and before the study proper), we also performed a reproducibility (test-retest) study of the aerobic PA and resistance training TPB measures.

#### Cognitive interviews for development of questions related to resistance training

Pre-tests of the resistance training component of the instrument was conducted with an independent sample of 7 women and 5 men (mean age = 67.6 years, SD = 8.2; and the mean age diagnosed with diabetes = 55.5 years, SD = 10.9). Participants were asked to complete the questionnaire and provide feedback on content applicability and grammar during in-depth interviews which ranged between 30 to 60 minutes in duration.

#### TPB measures for aerobic PA and resistance training (Study Proper)

The TPB constructs were measured using either 5- or 7-point Likert-type questions, and parallel items were used for both aerobic PA and resistance training. For the TPB questions, regular aerobic activity was defined as "150 minutes per week or more at a moderate intensity during your free time" and regular resistance activity was defined as "engaging in resistance training 3 times a week or more." These definitions are based on the Canadian Diabetes Associations guidelines for aerobic PA and resistance training [1].

Attitude towards regular aerobic and resistance activity was assessed using two sub-dimensions of instrumental (i.e., beneficial-harmful) and affective (i.e., enjoyable-unenjoyable) attitudes. The response format was a series of 7-point scales (1,7 = extremely, 2,6 = quite, 3,5 = slightly) and the phrases that preceded these items were: (1) "For me, meeting the guidelines for aerobic activity over the next 3 months would be/is..." and (2) "For me meeting the guidelines for strength training over the next 3 months would be/is...". The correlations (based on study proper) between the two attitude items for aerobic PA and resistance training were 0.57 (p < .001) and 0.62 (p < .001) respectively.

Subjective norm was measured with four items tapping the injunctive component and three items assessing the descriptive component [47,48]. The items were scored on 5-point scales ranging from 1 (strongly disagree) to 5 (strongly agree). Each of the subjective norm items were asked specifically for i) aerobic PA and ii) strength training. The injunctive items were: (1) "Most people in my social circle want me to meet the guidelines," (2) "Most people in my social circle would approve if I met the guidelines," (3) "My doctor or health care provider wants me to meet the guidelines," and (4) "My doctor or health care provider would approve for me to meet the guidelines" ('social circle' was defined as family and friends). The descriptive items were: (1) "Most of my family members achieve the guidelines," (2) "Most of my friends achieve the guidelines," and (3) "My spouse/partner achieves the guidelines." Cronbach's alpha coefficients for internal consistency (based on study proper) were 0.84 and 0.71 respectively for aerobic PA injunctive and descriptive norms; and 0.88 and 0.77 respectively for resistance training injunctive and descriptive norms.

Perceived behavioral control was measured each for aerobic PA and resistance training. The items were, (1) "Whether or not I participate in (i) aerobic/(ii) strength training is mostly up to me [49]." The items were scored on 5-point scales ranging from 1 (strongly disagree) to 5 (strongly agree).

Behavioral intention was assessed with four items for both aerobic and strength training [47]. Participants were asked: (1) "Based on the definition above, how motivated are you to meet the Guidelines for (i) aerobic activity/(ii) strength training over the next 3 months," from 1 (extremely unmotivated) to 7 (extremely motivated), (2) "How committed are you to meeting the Guidelines for (i) aerobic activity/(ii) strength training over the next 3 months," from 1 (extremely uncommitted) to 7 (extremely committed), (3) "How motivated are you to increase the amount of (i) aerobic activity/(ii) strength training that you are currently doing over the next 3 months?" from 1 (extremely unmotivated) to 7 (extremely motivated) and, (4) "I strongly intend to do everything I can to meet the Guidelines for (i) aerobic activity/(ii) strength training over the next 3 months" from 1 (extremely untrue) to 7 (extremely true). Cronbach's alphas (based on study proper) were 0.92 and 0.97 for aerobic and strength training respectively.

#### Test-retest study

A reproducibility study was conducted with an independent convenience sample of 26 participants. 11 women and 15 men initially completed the self-report TPB measures, and again two weeks later. Test-retest results (intraclass correlations, ICC) were excellent for all the TPB measures with the exception of the aerobic PBC result which was deemed fair [50]. The ICC's for aerobic PA were significant for attitude (r = 0.91, p < .001), injunctive norm (r = 0.89, p < .001), descriptive norm (r = 0.85, p < .001), PBC (r = 0.52, p < .05,) and intention (r = 0.94, p < .001). The ICC's for resistance training were also significant (ps' < .001) for the TPB constructs: attitude (r = 0.84), injunctive norm (r = 0.86), descriptive norm (r = 0.78), PBC (r = 0.90), and intention (r = 0.95).

### Data Analyses

For our primary objective, path analyses were conducted separately for aerobic PA and resistance training. Simultaneous multiple regression analysis was used to determine the associations of the TPB variables with behavior. Values for the aerobic PA and resistance training behavior variables were truncated to 3.29 SD from the mean to reduce the impact of outliers [51]. The relative contributions of the TPB variables (i.e., attitude, injunctive norm, descriptive norm, PBC and intention) were adjusted for age and gender. The TPB constructs were also tested to predict intention for both aerobic PA and resistance training.

To test our secondary objective, paired sample t-tests were conducted to compare means of the TPB constructs for both aggregated- and individual-item levels for the two modes of behavior (i.e., aerobic PA versus resistance training). SPSS (version 12.0 for windows) was used for all analyses.

## Results

**Figure 1:**
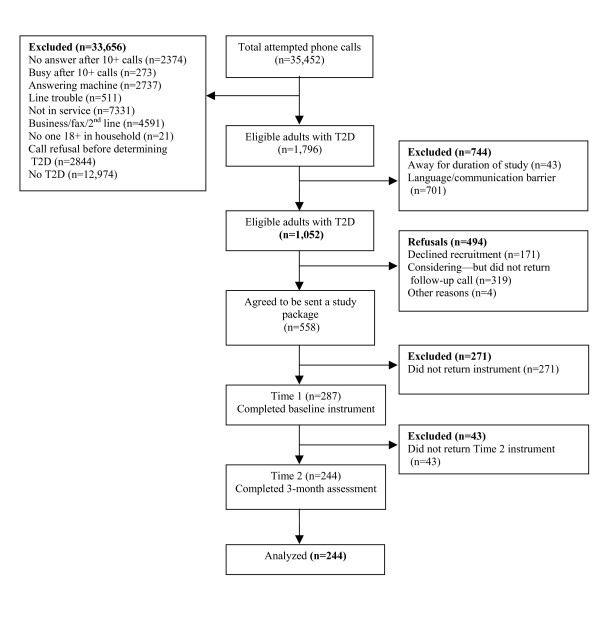
Study flow diagram for participant recruitment.

The flow of participants through the study is depicted in Figure 1. Calls were made to 35,452 generated numbers, which identified 1,796 eligible study participants (eligibility criteria included a diagnosis of T2D, being over the age of 18 years, and ability to complete a questionnaire in English). From this number (1,796), 558 individuals with T2D indicated they would be interested in receiving an information package (a priori quota of 558 individuals was established). The recruitment breakdown was as follows: British Columbia, n = 86; Prairie Provinces, n = 107; Central Canada, n = 317; and Atlantic Provinces, n = 48. 287 out of the 1,052 (27.3%) known eligible individuals with T2D completed and returned the package. 244 out of 1,052 (23.2%) individuals who completed the baseline questionnaires also completed and returned the time 2 questionnaires, yielding a response rate of 43.7% (244/558) of those initially recruited into the study.

The demographic, health and medical characteristics of the participants are displayed in Table 1. The demographic characteristics of our study generally reflect Canada's diabetic population in terms of age and sex distributions [52]. Canadians with T2D are older (majority over 65 years and older) with 54% being male [52]. Dropouts (n = 43) between the baseline and 3-month assessments revealed no significant differences for age, gender, ethnicity, marital status, education, income, health and medical characteristics (ps' > .05). However, a significant difference was found for age diagnosed with diabetes (p < .05). Dropouts were younger (M = 47.90, SD = 13.55) than individuals who stayed in the study until time 2 (M = 51.95, SD = 11.94).

**Table 1:** Demographic and Health Characteristics of Participants

	T2 total (n = 244)	
	
Variable	M (SD)	n (%)
Age	60.93 (11.23)	--
Age Diagnosed with Diabetes	51.95 (11.94)	--
Godin LTQ (minutes)	19.45 (21.47)	--
Meeting Guidelines for Aerobic PA		57 (23.5)
Meeting Guidelines for Resistance Training		41 (17.0)
Sex		
Male	--	131 (53.7)
Female	--	112 (45.9)
		
Ethnicity		
Canadian	--	183 (75.0)
Arab	--	2 (0.8)
African	--	1 (0.4)
European	--	32 (13.1)
Asian	--	15 (6.1)
Aboriginal	--	1 (0.4)
Latin, South American	--	0 (0)
Other	--	9 (3.7)
		
Marital Status		
Never married	--	10 (4.1)
Common law	--	11 (4.5)
Separated/Divorced	--	30 (12.3)
Married	--	171 (70.1)
Widowed	--	22 (9.0)
		
Education		
Some grade school	--	13 (5.3)
Some high school	--	37 (15.2)
Completed high school	--	41 (16.8)
Some university/college	--	38 (15.6)
Completed university/college	--	53 (21.7)
Some grad school	--	4 (1.6)
Completed grad school	--	21 (8.6)
Some technical training	--	14 (5.7)
Completed technical training	--	23 (9.4)
		
Gross Family Income		
< 20 000	--	42 (17.2)
20 000–39 999	--	73 (29.9)
40 000–59 999	--	56 (23.0)
60 000–79 999	--	30 (12.3)
80 000–99 999	--	15 (6.1)
> 100 000	--	22 (9.0)
		
Health Conditions		
Angina	--	31 (12.7)
Heart Attack	--	23 (9.4)
Stroke	--	9 (3.7)
Cancer	--	14 (5.7)
High Cholesterol	--	163 (66.8)
High Blood Pressure	--	160 (65.6)
Type 2 Diabetes	--	240 (98.4)
None	--	1 (0.4)
		
Smoking Status		
Regular Smoker	--	32 (13.1)
Occasional Smoker	--	7 (2.9)
Ex Smoker	--	105 (43.0)
Non Smoker	--	97 (39.8)
		
Medications		
Diabetes	--	198 (81.1)
Insulin	--	40 (16.4)
Pills	--	174 (71.3)
Cholesterol	--	140 (57.4)
Blood Pressure	--	158 (64.8)
		
Good or better health	--	165 (67.6)
		
Internet Access		
Yes	--	149 (61.1)
No	--	72 (29.5)

*Note*. Dashes signify not applicable.

Table 2 presents the bivariate correlations of the TPB and behavior measures. In our sample, 57 (23.5%) participants were meeting the recommended guidelines established by the Canadian Diabetes Association for aerobic PA (i.e., 150 minutes of moderate PA/week), while 115 (47.3%) were not participating in any form of aerobic PA (i.e., 0 days/week). Further, only 41 (17.0%) participants were meeting the recommended guidelines for resistance training (i.e., = 3 times a week), while 186 (76.9%) of the sample were not participating in any resistance training (i.e., 0 days/week).

**Table 2:** Bivariate Correlations of TPB Variables for Aerobic PA/Resistance Training

	1	2	3	4	5	6
1. Attitude	1					
2. Inj. Norm	0.40**/0.54**	1				
3. Des. Norm	0.22**/0.42*	0.51**/0.63**	1			
4. PBC	0.28**/0.35**	0.32**/0.47**	0.11/0.25**	1		
5. Intention	0.53**/0.62**	0.52**/0.47**	0.36**/0.48**	0.22**/0.24**	1	
6. Behavior T2	0.28**/0.27**	0.20**/0.26**	0.09/0.23**	0.05/0.09	0.29**/0.27**	1

** Correlation is significant at the 0.01 level (2-tailed).

* Correlation is significant at the 0.05 level (2-tailed).

**Figure 2:**
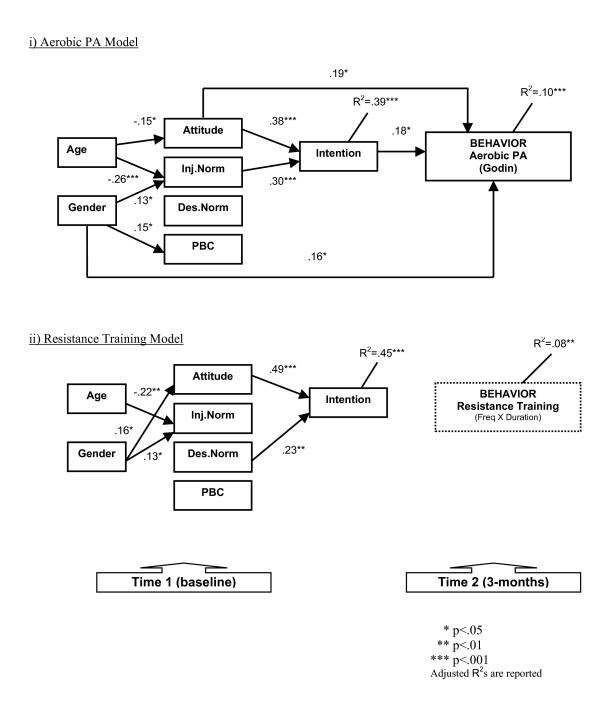
TPB path model showing the significant standardized beta coefficients of the background variables and TPB constructs.

In terms of our primary objective, attitude (β = 0.19), intention (β = 0.18), and gender (β = 0.16) were significantly associated with aerobic PA, while attitude (β = 0.38) and injunctive norm (β = 0.30) were significantly associated with intention. Age had significant associations with attitude (β = -0.15) and injunctive norm (β = -0.26), whereas gender had significant relationships with injunctive norm (β = 0.13) and PBC (β = 0.15). The model explained 10 and 39 percent of the variance respectively for PA behavior and intention (see Figure 2).

Results from the simultaneous multiple regression revealed that there were no significant associations with the TPB variables and resistance training behavior; although the model explained 8% of the variance (based on adjusted R-square value) in behavior. However, the model explained 45% of the variance for resistance training intention with attitude (β = 0.49) and descriptive norm (β = 0.23) being associated with intention. Age was significantly related with injunctive norm (β = -0.22), whereas gender was significantly associated with attitude (β = 0.16) and injunctive norm (β = 0.13) (see Figure 2).

Table 3 (Study Objective 2) provides the means and results of the paired sample t-test of the aerobic and resistance training TPB variables. Significant differences were found between the TPB constructs for the aggregated- and item-level measures for both aerobic PA and resistance training. All the aerobic PA mean scores for the aggregated- and item-level scores were significantly higher (ps' < .001) than the resistance training measures.

**Table 3:** Paired Sample t-test for TPB variables with Aerobic PA and Resistance Training

	Aerobic PA	Resistance Training		
	
Variables	M (SD)	M (SD)	t	p-value
*Aggregated-level*				
				
Attitude	5.19 (1.16)	4.44 (1.43)	8.24	< 0.001
Injunctive Norms	3.70 (0.89)	3.28 (1.03)	7.71	< 0.001
Descriptive Norms	2.90 (0.87)	2.64 (0.92)	6.51	< 0.001
PBC	4.22 (0.89)	4.05 (1.09)	3.65	< 0.001
Intention	4.62 (1.33)	3.87 (1.61)	8.25	< 0.001
				
*Item-level*				
Attitude				
Enjoyable	4.97 (1.35)	4.17 (1.59)	8.14	< 0.001
Beneficial	5.38 (1.31)	4.73 (1.56)	7.20	< 0.001
				
Subjective Norm				
Social circle wants	3.11 (1.29)	2.76 (1.25)	6.42	< 0.001
Social circle approves	3.50 (1.20)	3.11 (1.24)	6.86	< 0.001
Doctor wants	3.93 (1.03)	3.46 (1.20)	7.14	< 0.001
Doctor approves	4.15 (0.84)	3.71 (1.13)	7.06	< 0.001
Family behavior	3.05 (1.20)	2.81 (1.22)	5.44	< 0.001
Friends behavior	2.72 (1.00)	2.49 (1.02)	5.43	< 0.001
Spouse behavior	2.65 (1.26)	2.22 (1.18)	6.75	< 0.001
				
PBC				
Participation is up to me	4.22 (0.89)	4.05 (1.09)	3.65	< 0.001
				
Intention				
Motivation	4.51 (1.57)	3.83 (1.70)	6.53	< 0.001
Commitment	4.58 (1.48)	3.90 (1.66)	6.33	< 0.001
Increase 3 months	4.64 (1.35)	3.80 (1.64)	8.82	< 0.001
Intention	4.74 (1.48)	3.87 (1.72)	8.42	< 0.001

*Note*. All TPB constructs were measured using 5-point scales with the exception of attitude and intention which were 7-point scales.

## Discussion

This study tested the TPB in explaining aerobic PA and resistance training in a national population sample of T2D adults, and compared the mean scores of the TPB constructs between these two modes of PA.

The results from our path analyses provide partial support of the TPB's utility in predicting PA intention and behavior of T2D adults (main study objective). With aerobic PA, significant associations were found with attitude, intention, and gender explaining 10% of the variance with behavior. Relationships between aerobic intention with attitude and injunctive norm were also reported explaining 39% of the variance for intention. With resistance training, no significant associations with the TPB variables were reported with behavior, but the variables explained 8% of the variance. However, relationships existed between intention with attitude and descriptive norm, explaining 45% of the variance with resistance training intention. Age and gender differently affected the TPB constructs in both aerobic PA and resistance training.

Limited research has been conducted using the TPB to determine its predictive ability in explaining PA behavior in T2D. A study by Plotnikoff et al. [34], investigated the utility of the TPB in understanding aerobic PA in a large adult population with T1D (N = 697) and T2D (N = 1614). For both diabetes types, attitudes, subjective norms, and PBC were all significantly associated with intention, and intention was significantly associated with behavior [34]. Similar to that study, we found attitude and injunctive norm to be significantly associated with intention, and intention was significantly associated with aerobic PA. The magnitude of these relationships are also congruent with previous PA results from meta-analyses conducted [32,33], where attitude appeared to be the strongest correlate of intention, closely followed by PBC and with subjective norm being the weakest predictor.

In addition, the variances explained by the TPB for aerobic PA in our study were consistent with previous TPB studies as reported in meta-analytical studies [32,33]. The studies indicate that the TPB constructs explained approximately 30–46% of the variance for PA intention and 21–27% for PA behavior. The TPB's ability to explain behavior (10%) in our study was lower than that reported in previous TPB studies. This could be attributed to the unique study characteristics, the nature of the diabetes disease and related PA behavior. The adult diabetic population tends to be less active than the general population [4] most likely due to the physical limitations from the disease. Further, the majority of the TPB studies reviewed in the above meta-analyses employed cross-sectional designs [32,33].

As far as we know, our study is the first to examine the determinants of resistance training in individuals with T2D. It is also the first study to apply a theory-driven approach, namely the TPB, in examining the determinants of resistance training in a T2D sample. However, there is a small but emerging literature on the predictors of resistance training in non-T2D adults. Dean et al. [40] examined the efficacy of the TPB in understanding the factors influencing older adults' participation in strength training through purposeful sampling (N = 200) of men and women age 55 years and older from seniors' centers. Cross-sectional results revealed that subjective norm and PBC, but not attitude, explained 42% of the variance in strength-training intention, while intention, but not PBC explained 40% of the variance in strength-training behavior. Consistent with our study, descriptive norm significantly explained 45% of the variance in resistance training intention, but it was the addition of attitude, and not PBC, that contributed to the explained variance of the total TPB model. With regards to resistance training behavior, our study revealed no significant associations with intention or PBC with behavior. These inconsistent findings may be due to the different types of samples employed in the two studies, where Dean et al. [40] employed a non-diabetic/chronic disease sample.

Jette et al. [41] identified factors associated with resistance training exercise participation and adherence in a sample of sedentary, functionally limited, community-dwelling adults aged 60 to 94 years (N = 102) who were part of a 26-week home-based resistance training program. Cross-sectional findings of this older population study revealed that predictors of the frequency of exercise participation (number of exercise sessions performed divided by number of exercise sessions possible) were not the same as those that predicted high levels of adherence (number of calendar periods that participants exercised at least half the number of desired sessions) to the home-based program. Those participants with higher functional mobility, weaker muscle strength, and fewer new medical problems during the intervention participated more frequently in the home-based strength training program. None of the demographic factors, comorbidities, or psychological factors were significant predictors of participation. However, participants' positive attitudes toward exercise and strong sense of control over exercise, lower levels of perceived confusion, and depressed moods were associated with higher adherence to their individual home exercise programs. Although physical health variables were the primary indicators of overall participation in the program, it was the psychological factors that were most important to adherence to the home-based program [41]. Although our study did not measure adherence to resistance training, similar with the findings of Jette et al. [41], our results did reveal that attitude had a significant association with intention.

Further, Bryan and Rocheleau [39] found that all the TPB constructs were strongly associated with intention, and PBC was associated with resistance training behavior among college students (N = 210), of which 70% of the convenience sample was female. In our study, only attitude and descriptive norm were associated with intention, and none of the TPB variables had a direct effect on resistance training behavior. In addition, the TPB variables in Bryan and Rocheleau's [39] study, extroversion and perceived health accounted for 19% of the variance in aerobic exercise, while accounting for 40% of the variance in resistance training. Our study revealed 10% of the variance accounted for with aerobic PA behavior and 8% of the variance with resistance training behavior. It is important to note that these inconsistent results may be due to the differences in the study design and sample. Bryan and Rocheleau's [39] sample of college students were younger and disease-free, included other variables (i.e., extroversion and perceived health) in the TPB model, and employed an aggregated subjective norm measure.

The only study to present tests of the predictive validity of the TPB for aerobic-exercise behavior and resistance training was conducted among a convenience sample of young, healthy students [39]. An objective of the study examined whether extroverted personality and perceived health can be embedded in the TPB structure to improve the specificity of the model for exercise behaviors. The results revealed that the TPB constructs of attitude, norms, and PBC exhibited strong correlations with aerobic intentions, and PBC had a significant direct effect on aerobic behavior. Contrary to these findings, our study revealed that only attitude and injunctive norm exhibited associations with aerobic intention, and it was attitude that had a significant direct effect on aerobic behavior.

Age and gender in our study were found to act differently on the TPB constructs in both aerobic PA and resistance training. In our study, being younger and of the male gender were associated with higher mean scores in the TPB constructs. It is important to differentiate between age and gender when examining any population, as age and gender can be important determinants of PA [53]. For example, Bryan and Rocheleau [39] note that individuals who engage in aerobic activity versus resistance training often have different goals, and the difficulty of performing these two activities may be quite different.

In regards to resistance training, there was no significant relationship found between intention and behavior in our study. However, the bivariate correlation did reveal that resistance training intention had a significant association with resistance training behavior (r = 0.27, p < .01). Other TPB variables including attitude (r = 0.27, p < .01), injunctive norm (r = 0.26, p < .01), and descriptive norm (r = 0.23, p < .01) were also significantly correlated with resistance training behavior. Although injunctive norm (β = 0.14) and resistance training intention (β = 0.12) contributed to the 8% of explained variance for resistance training in the multiple regression model, neither variable reached significance at the .05 level. Relatively consistent with Plotnikoff [4] who reported only 12% of a large T2D population sample of adults performing any form of resistance training, our study reported 23% of the sample engaging in this behavior (of which only 17% were meeting guidelines). This may have limited the statistical power in our analyses to examine the determinants of this behavior.

Further, given the literature to explain resistance training is relatively embryonic, we conducted a set of additional analyses with different classifications of the resistance training dependent measure. These included multiple regression analyses using (1) frequency only (i.e., number of times individuals reported engaging in resistance training) which explained 4% of the variance; and (2) employing three resistance training categories [not engaging in any resistance training, engaging in some resistance training (i.e., 1–2 times/week), and meeting resistance training guidelines (i.e., = 3 times/week)] which accounted for 6% of the behavior. Two sets of logistic regression analyses also examined (1) those meeting (i.e., = 3 times/week) *versus *not meeting guidelines (i.e., < 3 times/week); and (2) those engaging in any resistance training (i.e., > 0 times/week) *versus *not engaging at all in this behavior (i.e., 0 times/week), explaining 11% and 16% of the variance respectively. However, none of these additional analyses produced any significant associations between the TPB variables with resistance training behavior.

Nevertheless, the TPB does hold partial utility for resistance training intention which is consistent with previous studies [39,40]. Resistance training is a relatively novel behavior in the T2D population where the intention-behavior gap may be a realistic indication of how psychosocial cognitive factors directly influence intention, but does not necessarily translate into behavior. There may be other factors (e.g., lack of experience, lack of knowledge) for both aerobic PA and resistance training which may have impacted this relationship that were not examined in our study. Also, it is not surprising that many of the social cognitive measures used to predict general PA are more relevant for aerobic activity since the majority of exercisers are engaging in only aerobic forms of activity [4]. For example, PBC and attitude towards an aerobic activity such as walking, may be quite different from the barriers, perceived control and attitudes towards resistance training. Resistance training may be daunting for some and may be influenced by control factors including access to facilities and special equipment, and knowledge about what exercises to perform [4,40]. Testing the TPB with additional constructs [e.g., environmental factors (costs, equipment/facilities), observational learning, behavioral capabilities (skill acquisition)] may further help to explain this behavior.

The inclusion of resistance training behavior in the TPB model still remains exploratory. Until resistance training behavior becomes more widely engaged in the population, the application of social cognitive models (including the TPB) cannot be fairly assessed at this point. Theoretical research is also needed on the intention-behavior gap with resistance training to determine the most salient predictors of this behavior to guide interventions by operationalizing appropriate theoretical constructs.

In conjunction with the consistent findings of the TPB in predicting PA behavior, significant differences were also found in the means and trends between aerobic PA and resistance training at both the global- and item-level of the TPB constructs (our second study objective). All the means for aerobic PA were significantly higher compared to the means for resistance training. This suggests the importance of how the relationships between the TPB variables vary in different PA settings. Bryan and Rocheleau [39] found that the TPB model for resistance training had greater predictability than that of aerobic exercise, with more variance explained for resistance training. The TPB's ability to account for a greater proportion of variance in resistance training was attributed to the stronger role of PBC. In other words, resistance training appears to be more strongly influenced by volitional control than aerobic activity due to increased equipment and training required, as well as knowledge needed to engage in it. Thus, PBC becomes a more significant direct predictor of resistance training.

Overall, our study results provide partial evidence towards the utility of the TPB for practitioners and researchers to develop and evaluate appropriate PA interventions for populations with T2D. While TPB appears to have good utility in predicting both aerobic and resistance training intention, its predictive ability was less evident for behavior in both modes of exercise. According to Ajzen [54], behavioral intentions must attempt to influence the beliefs that ultimately lead to the performance of the behavior. Fishbein, Von Haeften, and Appleyard [55] advocate identifying salient beliefs from the target population, developing persuasive messages around the beliefs, and then developing appropriate material based on the elicited beliefs. Based on our study results, these interventions would need to apply strategies for increasing the salience of attitude and descriptive norm for resistance training intention. For aerobic PA, specific emphasis should be placed on enhancing positive attitudes towards PA and having important others approve the PA behavior. On the other hand, specific emphasis should be placed on the importance of social norms and enhancing positive attitudes towards PA in interventions using resistance training. For example, for increasing the salience of attitude, interventions may focus on highlighting the benefits and enjoyment aspects of both aerobic PA and resistance training. For social norms, interventions may include messaging materials that encourage individuals with diabetes to perform aerobic PA and resistance training with a friend.

However, this study needs to be interpreted within the context of its limitations. The socio-cognitive and PA measures relied on self-report which can introduce measurement error such as recall error, social desirability and other reporting biases. Future research should use objective measures for assessing aerobic PA (i.e., pedometry, accelerometry) and consider employing validation studies (e.g., observation techniques) for resistance training measures. In addition, our study results need to be treated with some caution in terms of their generalizability to the national T2D population given the relatively low response rate. It may be that the respondents were more motivated for activity which may have led to an over-assessment of the true, more general predictive value of TPB variables for both aerobic PA and resistance training.

In conclusion, this study adds to the limited literature base on the TPB and aerobic PA in T2D, as there is currently only one study that has examined aerobic PA predictors in TPB [34]. We employed a national, random sample which is an additional strength in our study. Our study provides the first test of the TPB in the PA domain on a diabetic population, simultaneously examining the psychosocial cognitive factors (with parallel items) that influence both aerobic PA and resistance training. Also, it is the first of any social cognitive theory examining predictors of resistance training in this population. Further, the test-retest component in the study design added to the scale reliability of the cognitive measures. Since resistance training is relatively novel in the PA and diabetes literature, future research is needed to examine other predictors that may be present in understanding resistance training to further guide, develop, and evaluate theory-based interventions in this population. Future research is also warranted to broaden the TPB beyond its existing social-cognitive constructs by including social and environmental factors [56] for both aerobic PA and resistance training in this population.

## Competing interests

The authors declare that they have no competing interests.

## Authors' contributions

RCP conceived of the study, and participated in its design and coordination and helped to draft and revise the manuscript. KSC, LT and RJS have been involved in drafting the manuscript and revising it critically for important intellectual content. NK performed and interpreted the statistical analysis, as well as helped to draft the manuscript. All authors read and approved the final manuscript.
